# Breast pericytes: a newly identified driver of tumor cell proliferation

**DOI:** 10.3389/fonc.2024.1455484

**Published:** 2024-12-17

**Authors:** Katelyn Del Toro, Yamhilette Licon-Munoz, William Crabtree, Tristan Oper, Christine Robbins, William C. Hines

**Affiliations:** Department of Biochemistry and Molecular Biology, University of New Mexico School of Medicine, 1 University of New Mexico MSC08 4670, Albuquerque, NM, United States

**Keywords:** breast pericytes, vascular smooth muscle cells, perivasculature, breast cancer, single-cell RNA-sequencing (scRNA-seq), primary culture

## Abstract

**Introduction:**

Effective treatment of breast cancer remains a formidable challenge, partly due to our limited understanding of the complex microenvironmental factors that contribute to disease pathology. Among these factors are tissue-resident perivascular cells, which play crucial roles in shaping vascular basement membranes, maintaining vessel integrity, and communicating with adjacent endothelial cells. Despite their essential functions, perivascular cells have been relatively overlooked. Identifying them by immunostaining has been challenging due to their low abundance, inherent heterogeneity, and shared marker expression with other cell types. These challenges have hindered efforts to purify pericytes and generate primary cell models for studying their biology.

**Methods:**

Using a recently developed FACS method, we successfully identified and purified each cell type from breast tissues, allowing us to deep-sequence their transcriptomes and generate primary cell models of each cell type—including pericytes. Here, we used these data to analyze cell-type-specific gene expression in tumors, which revealed a strong association between pericyte-specific genes and breast cancer patient mortality. To explore this association, we defined the heterogeneity of breast pericytes using single-cell RNA sequencing and identified a broad marker for visualizing perivascular cells in breast tumors.

**Results:**

Remarkably, we discovered perivascular cells dissociated from vessels and emerged as a dominant mesenchymal cell type in a subset of breast tumors that contrasted with their normal perivascular location. Moreover, when we purified pericytes from the breast and cultured them alongside breast tumor cells, we discovered that they induced rapid tumor cell growth significantly greater than isogenic fibroblast controls.

**Discussion:**

These findings identify perivascular cells as a key microenvironmental factor in breast cancer, highlighting the critical need for further research to explore their biology and identify specific stimulatory mechanisms that could be targeted therapeutically.

## Introduction

**Figure 1:**
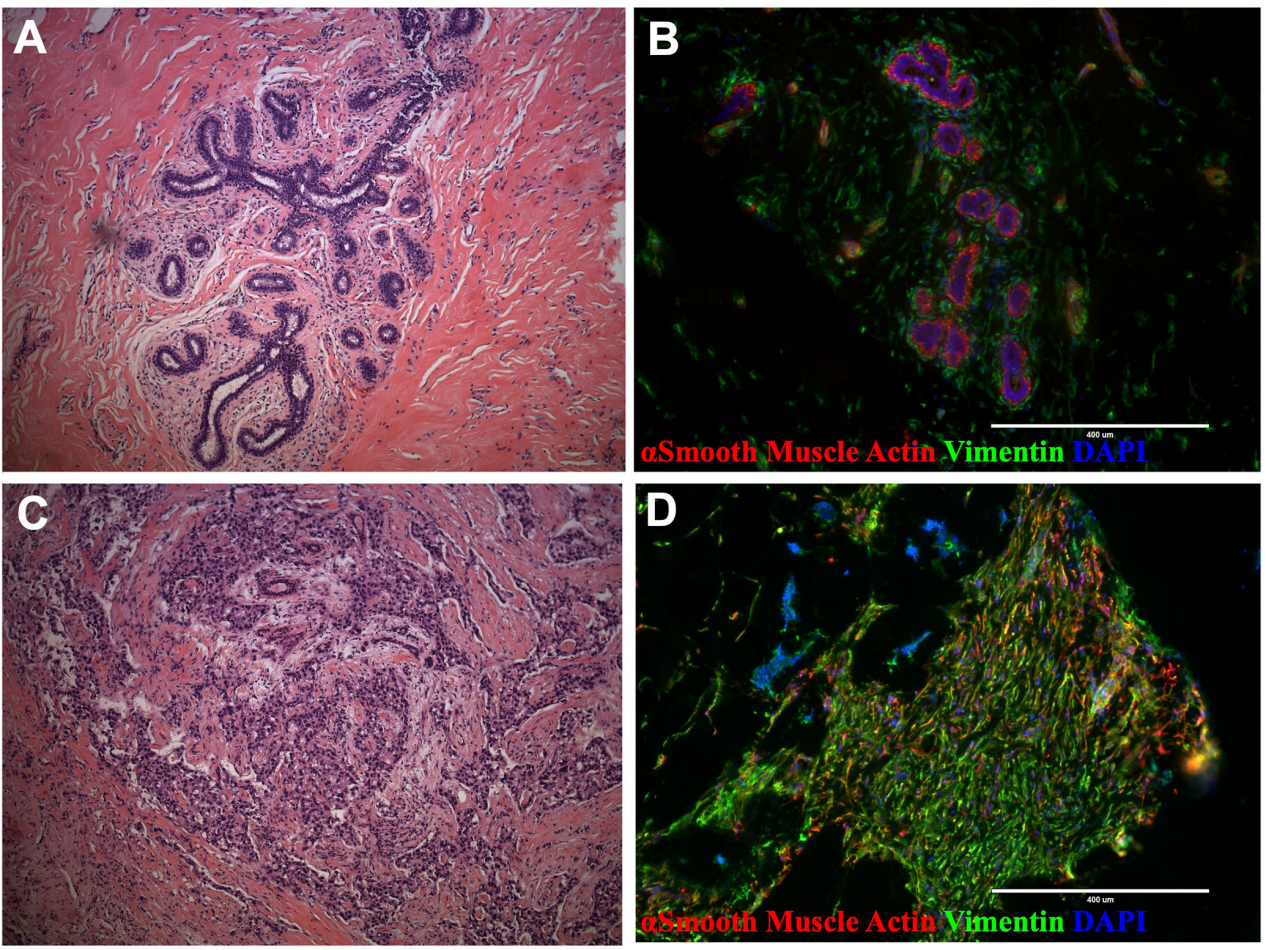
Breast tissue and tumor architecture. **(A)** H&E staining of normal breast tissue (21-year-old female) **(B)** Normal breast tissue immunostaining with actin (green) and vimentin (red; reduction mammoplasty, 18-year-old female). **(C)** H&E staining of malignant breast tumor (50- year-old female). **(D)** Tissue immunostaining of malignant breast tumor with actin (green) and vimentin (red; 33-year-old female).

The breast microenvironment is a complex network of cells and biomolecules that includes extracellular matrix proteins, glycoproteins, and signaling molecules derived from a cadre of epithelial, endothelial, mesenchymal, and a spectrum of immune cell types. These elements interact dynamically and reciprocally to maintain the tissue’s structure and function (1, 2). In invasive breast tumors, this delicate balance becomes disrupted (**Figures 1A–D**). Tumors typically exhibit activated fibroblasts, increased immune cell infiltration, and areas of angiogenesis (3–8). Moreover, among other changes, the basement membrane separating the epithelial and stromal compartments is often compromised or absent, facilitating direct interactions between epithelial tumor cells and stromal cells that typically would not occur (9–11).

**Figure 2:**
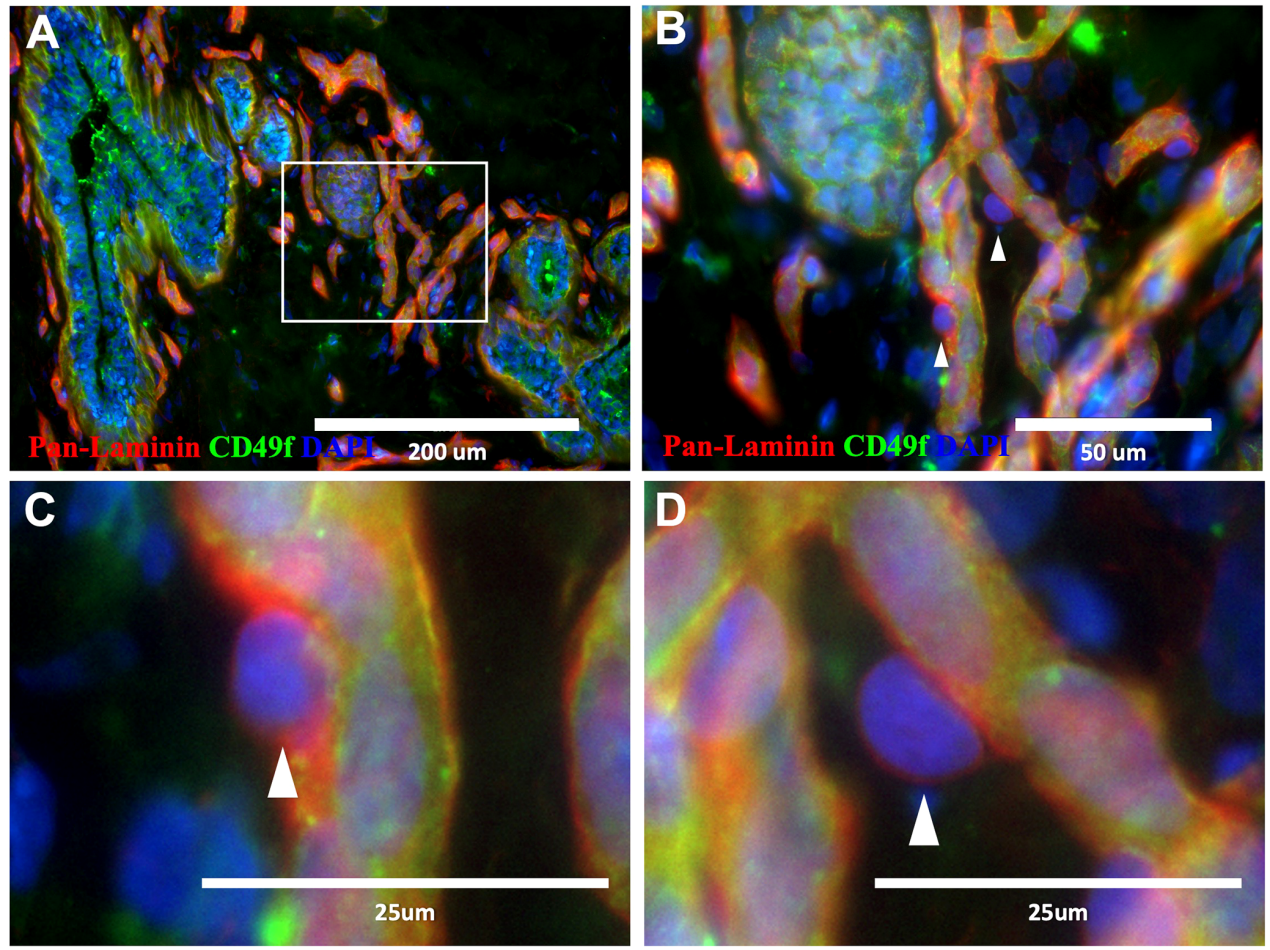
Pericytes in normal breast tissue. **(A–D)** Normal breast tissue immunostained with CD49f (green) and pan-laminin (red; 18-year-old female). Pericytes are present along the periphery of capillaries **(B–D)**, ‘▲’ symbol indicates select pericytes).

While tumor-associated fibroblasts, macrophages, and expanding blood vessels have been extensively studied for their roles in malignancy, other cell types, such as pericytes, have received far less attention (12). Pericytes are a subset of perivascular mesenchymal cells that envelop capillaries and small vessels (**Figure 2**). They are generally considered to serve auxiliary functions that contribute to the integrity of blood vessels and the blood-brain barrier (13–17). Pericytes are also involved in angiogenesis and immune cell activation (13–16, 18, 19). However, they have been relatively understudied outside the central nervous system (12), and whether they and other perivascular cells play a more direct role in tumor cell communication is unresolved. Technical challenges contribute to this limited research, as pericytes and other perivascular cells comprise a range of relatively unclassified subtypes, from vascular smooth muscle cells on larger vessels to the different types of pericytes along capillary processes (12, 20, 21). This variability in phenotype and marker expression makes identifying pericytes problematic (14, 22, 23). While there are a few markers for pericytes, their expression is heterogeneous, and some are expressed by other non-perivascular cell types, such as fibroblasts, leading to cell mischaracterizations (13, 14, 24). Thus, it has been challenging to identify, isolate, and create perivascular cell models to test their functional characteristics (14).

Using a combination of histological, cytometric, and molecular tools, we recently performed a comprehensive cellular dissection of the human breast and created cell models for every breast cell type, including pericytes (25). Transcriptome analysis of each FACS-purified cell type allowed us to develop a broad perspective of each cell type’s fundamental properties and individual functions (26). These data provided a strong foundation for exploring the roles of each cell type in normal breast tissue biology. However, their respective and emergent roles in the growing tumor organ remained a pressing question.

Here, we leveraged our transcriptomic data to investigate the enrichment of cell-specific genes within clinical tumors. After identifying a strong association between perivascular genes and patient mortality, we set out to uncover the underlying mechanisms driving this correlation. We identified a pan-pericyte marker using single-cell RNA sequencing, enabling us to visualize the perivascular cells within breast tumors. Remarkably, we found that pericytes had detached from blood vessels and became a predominant stromal cell type in a subset of tumors. Using our primary pericyte cell models, we assessed the growth impact of purified breast pericytes on tumor cells. Our findings revealed that pericytes substantially influence breast tumor cells, promoting remarkable growth that even surpassed isogenic, activated fibroblast controls.

The dramatic increase in tumor cell proliferation observed in the presence of pericytes, the detachment of perivascular cells from blood vessels, their significant presence within the tumor stroma, and their genes’ association with patient survival collectively suggest that they contribute to the supportive niche that facilitates tumor progression.

## Results

### Perivascular-specific genes are associated with reduced breast cancer survival

Breast tissues comprise at least twelve cell types with distinct functions mediated by their unique gene expression patterns (25). In our prior studies, we identified each breast cell type, then physically isolated, sequenced, and analyzed their transcriptomes (26). These breast cell types include two luminal epithelial types, myoepithelial cells, perivascular cells, lymphatic and vascular endothelial cells, fibroblasts, adipocytes, leukocytes, and a novel epithelial population. Furthermore, we developed cell models of essentially every cell type that now permits exploration of their functional characteristics.

**Figure 3:**
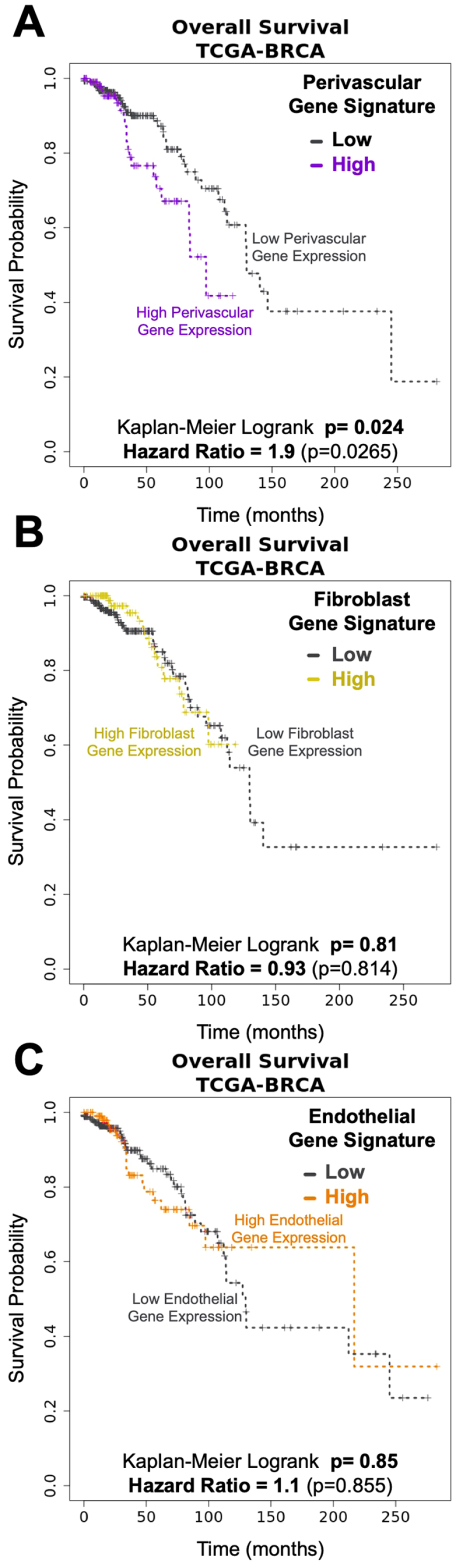
Pericytes gene signatures associated with Breast Cancer survival. Kaplan-Meyer curves of breast cancer survival (TCGA- breast cancer data set) associated with genes unique to breast **(A)** pericytes (log-rank: p= 0.024, HR=1.9), **(B)** fibroblasts (logrank: p=0.81, HR= 0.93), and **(C)** endothelial cells (log-rank: p=0.85, HR= 1.1).

Expanding on our earlier findings, we investigated the expression of cell-type-specific gene signatures in breast tumors (TCGA breast cancer cohort, **Supplementary Figure S1**) to explore each cell type’s potential involvement in tumor pathology. Remarkably—and in contrast to the other cell types, we found that the perivascular cell signature was associated with reduced patient survival (**Figure 3A**; p=0.024). Patients with an elevated perivascular signature had nearly twice the risk of death at any given time point compared to those with lower perivascular gene expression (**Figure 3A**, Hazard Ratio 1.9). This finding contrasts with the other stromal cell type signatures, which did not associate with patient survival, such as fibroblasts (**Figure 3B**; p=0.81, HR=0.93), adipocytes (**Supplementary Figure S2A**; p=0.061, HR=0.52), vascular endothelial cells (**Figure 3C**; p=0.85, HR=1.1), lymphatic endothelial cells (**Supplementary Figure S2B**, p=0.505, HR=0.8), and CD45+ leukocytes (**Supplementary Figure S2C**, p=0.1, HR=0.59).

The epithelial cell gene signatures were also not associated with survival. These include myoepithelial cells and ‘Pop4’ epithelial cells (25) (**Supplementary Figures S3A, B**; respectively, p=0.59, HR=0.84 and p=0.5, HR=0.8). Also not associated with survival was the gene signature derived from estrogen receptor (ER)-negative luminal cells—associated with the basal molecular subtype of breast cancer (27, 28) (**Supplementary Figure S3C**, p=0.19, HR=0.69). The ER+ luminal cell signature, which we predict would be aligned with the luminal molecular subtypes (29), had a trend of improved survival in the first few years after diagnosis – a previously reported feature of this clinical subtype (30–32) (**Supplementary Figure S3D**, p=0.111, HR=0.59). However, this early difference equalized over time, leading to no significant difference between patient groups (high vs. low ER+ luminal gene signature, **Supplementary Figure S3D**, p=0.111, HR=.59).

The above analyses reveal that perivascular cells may have a significant role in the pathology of malignant breast disease. Furthermore, the difference in associations between survival and the perivascular and endothelial cell signatures indicates that, in some tumors, the normal balance between perivascular cells and endothelial cells becomes disrupted.

### Identification of cyclic GMP-dependent protein kinase as a broadly expressed perivascular marker

**Figure 4:**
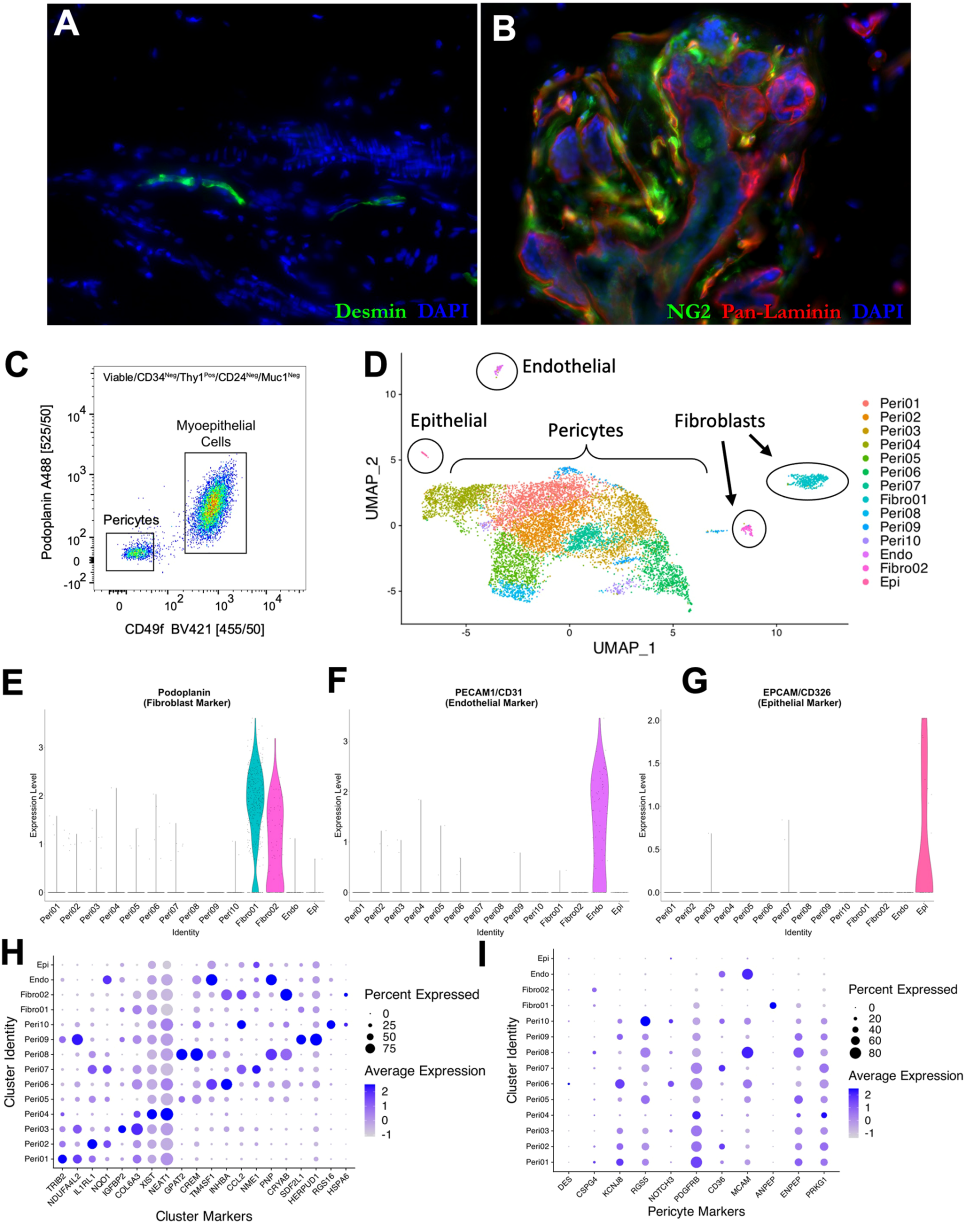
Pericyte heterogeneity. Normal breast tissue immunostaining with **(A)** desmin (green) and pan-laminin (red; 23-year-old female) and **(B)** NG2 (green) and pan-laminin (red; 33-year-old female. **(C)** Final FACS scatter plot of breast cells (33-year-old female) stained for CD49f (BV421) and Podoplanin (A488) to enrich for pericytes. The remaining gates are in **Supplementary Figure S4**. **(D)** Uniform manifold projection (UMAP) analysis of single-cell sequencing performed on an enriched breast pericyte population. Clusters of epithelial cells, endothelial cells, and fibroblasts are circled. Violin plots exhibiting expression of **(E)** podoplanin (fibroblast marker), **(F)** PECAM/CD31 (endothelial marker), and **(G)** EPCAM/CD326 (epithelial marker) in each single-cell sequencing cluster. Dot plots showing average expression levels of **(H)** unique markers to each single-cell sequencing cluster and **(I)** known perivascular markers.

To investigate the association between the perivascular cells and patient survival, we pursued visualizing these cells in clinical tumors. The lack of a specific and reliable marker posed a problem, however. Common markers, such as Desmin and NG2, often used to identify pericytes in normal tissues, are not always consistently expressed by these cells (**Figures 4A, B**), a finding that aligns with caveats described in prior reports (14, 33). In normal tissues, the identification of pericytes is aided by their histological location, as they envelope blood vessels and are embedded in the basement membrane. Given the chaotic nature of malignant tissues, this organization and basement-membrane compartmentalization is likely disrupted in tumors. Thus, a marker—that can reliably discriminate between all perivascular cells and fibroblasts—was needed.

To identify a differentially expressed marker, we sequenced FACS-enriched pericytes using single-cell RNA sequencing (**Figure 4C**; **Supplementary Figure S4**). Included in the sample was a limiting number of endothelial cells, epithelial cells, and fibroblasts that could serve as comparator controls. After sequencing, we identified 8,970 pericytes, 451 fibroblasts, 74 endothelial cells, and 26 epithelial cells via their expression of cell-type-specific genes (**Figure 4D**). A total of 21,288 genes were detected across all cell types, with a median of 1,514 genes detected per cell. We performed a non-linear dimensional reduction using the Uniform Manifold Approximation and Projection technique (UMAP) to explore the dataset and identify potential transcriptional differences between cells.

Our analysis resolved ten perivascular cell clusters, each identified by their distinct gene expression patterns (**Figure 4D**). The fibroblasts, endothelial, and epithelial cells were readily distinguished from pericytes due to their expression of cell-specific genes, including *PDPN* (podoplanin), *PECAM1*, and *EPCAM*, among others (**Figures 4E–G**). Differentially expressed genes characterizing the perivascular and other cell type clusters validated the algorithm’s settings and output (**Figure 4H**). Consistent with our prior tissue staining and other reports (14, 33), we found genes encoding frequently used pericyte markers to be unique to pericytes but expressed by only a small proportion of them (**Figure 4I**). For example, Desmin (*DES*) was expressed only by cells within pericyte cluster six—and only by a small fraction of these cells (<20%). Some markers were expressed by a small proportion of pericytes, but also by fibroblasts; for example, *CSPG4* (the gene that encodes the NG2 antibody epitope) was expressed in perivascular cluster eight –as well as both fibroblast clusters. Other markers were not uniformly expressed by all pericyte clusters –but were expressed by fibroblasts and/or other cell types; for instance, *PDGFRB* and *CD36* were expressed in fibroblasts and endothelial cells, respectively. Additionally, some markers were not expressed uniformly by all pericyte clusters; for example, *RGS5* is diminished in perivascular clusters five and six. The lack of uniformity and specificity of these markers presents a problem in tumors, where we cannot rely on histology and morphology to identify perivascular cells—which, in these cases, may no longer be ‘peri’-vascular.

**Figure 5:**
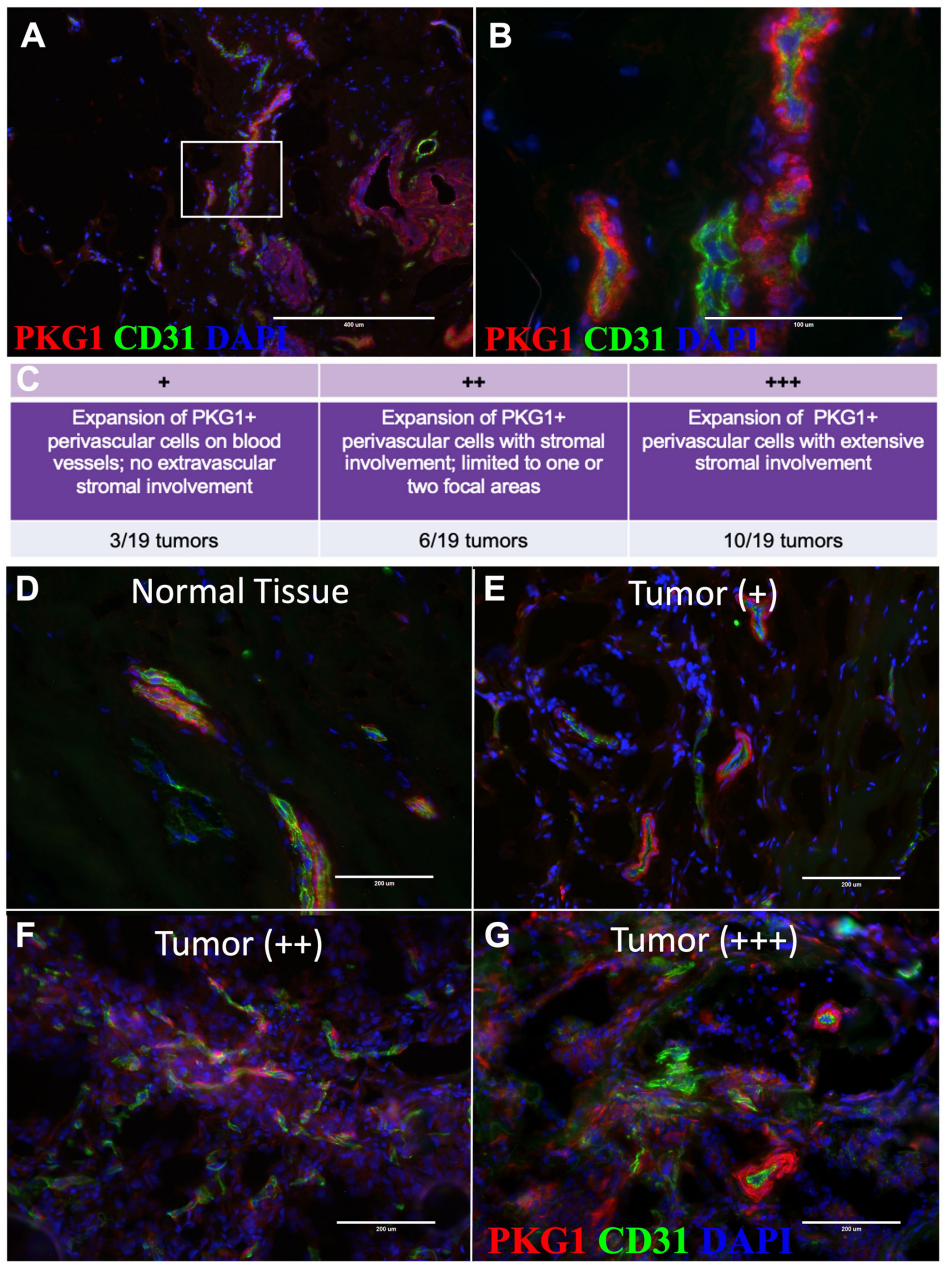
Pericytes expand into tumor stroma. **(A)** Tissue immunostaining of normal tissue with PKG1 (red) and CD31 (green) at 10X and **(B)** 20X (18-year-old female). **(C)** Grading system for characterizing pericyte expansion in tumors. **(D–G)** Representative images of tissue immunostaining with PKG1 (red) and CD31 (green). **(D)** Normal breast tissue (upper left), **(E)** + invasive breast tumor, **(F)** ++ invasive breast tumors, and **(G)** +++ invasive breast tumors are shown.

To identify a perivascular-cell marker that could readily distinguish perivascular cells from fibroblasts, we searched for genes differentially expressed by all pericyte clusters in the scRNA-seq dataset (compared to fibroblasts). The top genes best meeting these criteria included alpha 7 integrin (*ITGA7*), Cysteinyl Leukotriene Receptor 2 (*CYSLTR2*), GLIS Family Zinc Finger 2 (*GLIS2*), Pyruvate Dehydrogenase Kinase 4 (*PDK4*), Phosphatidylinositol Transfer Protein Cytoplasmic 1 (*PITPNC1*), Nuclear Receptor Subfamily 4 Group A Member 1 (*NR4A1*), and CGMP-Dependent Protein Kinase 1 (*PRKG1*, **Supplementary Table 1**). Antibodies for each protein were acquired and tested by immunostaining normal breast tissues (**Supplementary Table 2**). Lack of specificity and high background staining were common issues for every antibody except for the anti-Pkg1 antibody (specific to CGMP-Dependent Protein Kinase 1). When applied to tissues, the Pkg1 antibody stained the perivasculature intensely, identified by its circumscription of CD31-stained endothelium (**Figures 5A, B**; **Supplementary Figure S5A**). As expected by the gene expression data, we found pkg1 stained myoepithelial cells also, albeit at diminished levels. We moved forward and reasoned that myoepithelial staining would not pose an issue when staining tumors as long as we co-stained with an epithelial marker.

### Pkg1 tumor staining reveals stromal perivascular cell expansion

Remarkably, after applying the anti-pkg1 antibody to breast tumors (n=19, **Supplementary Table 3**), we observed significant perivascular cell expansion in nearly half of all breast tumors analyzed (**Figures 5C–G**). We confirmed that the pkg1 staining was specific to pericytes by co-staining with CD26, CD45, and EpCAM, which allowed us to exclude fibroblasts, leukocytes, and epithelial tumor cells, respectively (**Supplementary Figures S5B–F**). Typically, we saw pericyte expansion along the periphery of blood vessels and the extension of pkg1-stained cells into the tumor stroma, with the extent of involvement varying between individuals (**Figure 5E–G**).

We categorized the stained tumors based on their vessel and stromal involvement. The first group consisted of tumors where the Pkg1+ cells had expanded on blood vessels in at least one focal area, but extravascular stromal involvement was not observed (3/19 tumors, **Figures 5C, E**; ‘+ stained tumors’). The second group exhibited stromal expansion of Pkg1+ perivascular cells limited to only one or two focal areas (6/19 tumors; **Figures 5C, F**; ++ stained tumors’). The last group exhibited widespread involvement of the perivasculature, where Pkg1+ cells dominated the stromal compartment (10/19 tumors; **Figures 5C, G**; ‘+++ stained tumors’). These findings demonstrate that pericytes, normally confined to the blood vasculature, can expand on blood vessels in tumors, dissociate, and sometimes dominate the tumor stroma.

### Breast pericytes induce tumor cell proliferation

Macrophages and fibroblasts are two prominent stromal cell types known to enhance tumor cell growth (34, 35). Although mesenchymal, pericytes differ considerably from fibroblasts in their normal histological location, gene expression, and overall function (13, 14, 25, 26). However, whether breast pericytes possess similar tumor-promoting properties is unresolved—a knowledge gap partially resulting from a lack of available breast pericyte cell models.

In the normal breast microenvironment, epithelial cells are separated from perivascular cells by other cell types, the extracellular matrix, and both the epithelial and vascular basement membranes. These barriers are lacking or significantly fragmented in breast tumors (36). Thus, as demonstrated by our Pkg1 staining in tumors, there is potential for more intimate interactions between perivascular cells and tumor cells in a subset of breast malignancies, the ramifications of which are currently unknown (**Figures 5E–G**; **Supplementary Figure S5E**).

**Figure 6:**
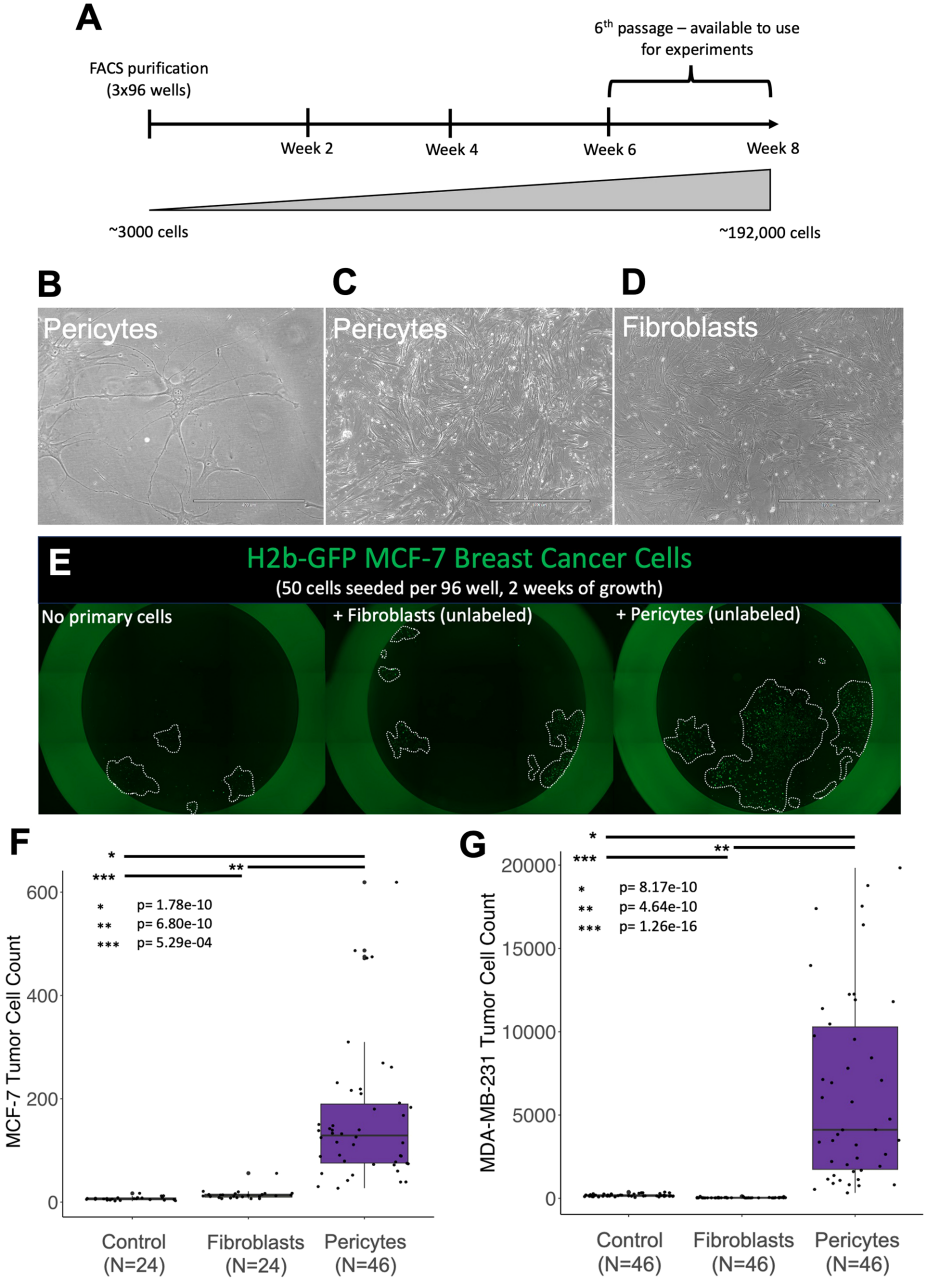
Pericytes induce rapid tumor cell proliferation. Phase images of normal breast pericytes— purified from tissue (37-year-old female) at **(A)** low confluency and **(B)** high confluency. **(C)** Phase image of normal breast fibroblasts at high confluency. **(D)** Timeline for cell expansion **(E)** Representative images of H2b-GFP-expressing MCF7 cells (green) co-cultured with primary breast cells in 1% serum Fluorobrite media (unlabeled fibroblasts or pericytes) **(F, G)** cell counts for H2b-GFP-expressing **(F)** MDA-MB-231 cells or **(G)** MCF-7 breast cancer cell lines co-cultured with primary breast pericytes or fibroblasts for two weeks. Control plates did not contain a primary cell line but the tumor cell line of interest. Relevant p-values are indicated by *, **, and *** (calculated one-tailed t-test, unequal variance).

To evaluate the potential contributions of pericytes to tumor cell growth, we used our novel primary pericyte cell models (FACS-purified from breast tissues) and co-cultured them with breast tumor cells. The stringent purification of primary breast pericytes—and fibroblast controls—was performed as previously described (25). After FACS purification, the cells were expanded for 6-8 weeks to obtain the required number of cells for co-cultures (**Figure 6A**). As expected, cultured pericytes exhibited a unique stellate morphology at low density but were virtually indistinguishable from fibroblasts at higher confluence (25) (**Figures 6B–D**). Because contaminating fibroblasts could potentially take over the pericyte cultures, monitoring cell identities was paramount. We accomplished this by immunostaining cultures for CD36, a marker that distinguishes cultured pericytes and fibroblasts (25) (**Supplementary Figures 6A–C**). Validated primary pericyte cultures, purified from different individuals, were used for all subsequent co-culture experiments.

We tested the functional activity of pericytes by culturing them with MCF-7 tumor cells—a luminal cell model of ER-positive breast cancer. After two weeks of co-culture, we imaged the plates, quantified the tumor cells, and discovered remarkable tumor cell expansion in the wells containing pericytes (**Figures 6E, F**). Compared to MCF-7 cells cultured in medium alone, those co-cultured with pericytes contained a striking 23.5-fold greater number of cells (**Figure 6F**; p=1.78e-10). Fibroblasts, used as a positive control, also increased MCF-7 growth but were not nearly as effective as pericytes, resulting in only a 2.12-fold increase compared to cells cultured in the medium alone. These experiments were performed under identical conditions in a 96-well dish using adjacent wells, ruling out the possibility of batch effects causing the observed differences.

The above experiment was performed using a cell model of luminal breast cancer, a phenotype that accounts for roughly 75% of clinical diagnoses. Basal breast tumors, characterized by their lack of estrogen and progesterone receptors while maintaining normal Her2 (*ERBB2*) levels, are another major clinical subtype. To determine whether the observed pericyte stimulation was limited to luminal MCF-7 cells or exhibited a broader effect across tumor cell types, we performed the co-culture experiment using the ‘Basal B’ (37) MDA-MB-231 tumor cell model. The results were consistent. We found that the faster-growing MDA-MB-231 cells cultured alongside pericytes also displayed explosive growth, containing, on average, 36-fold more cells than the cells grown in the medium alone (p=8.17e-10, **Figure 6G**). Interestingly, in contrast to co-cultures with MCF-7 cells, fibroblast co-cultures did not stimulate growth of MDA-MB-231 cells (p=1.26e-16, **Figure 6G**). These results indicate that pericyte stimulation is not limited to a particular single tumor cell line but represents a broader capability of pericytes to enhance tumor cell proliferation.

**Figure 7:**
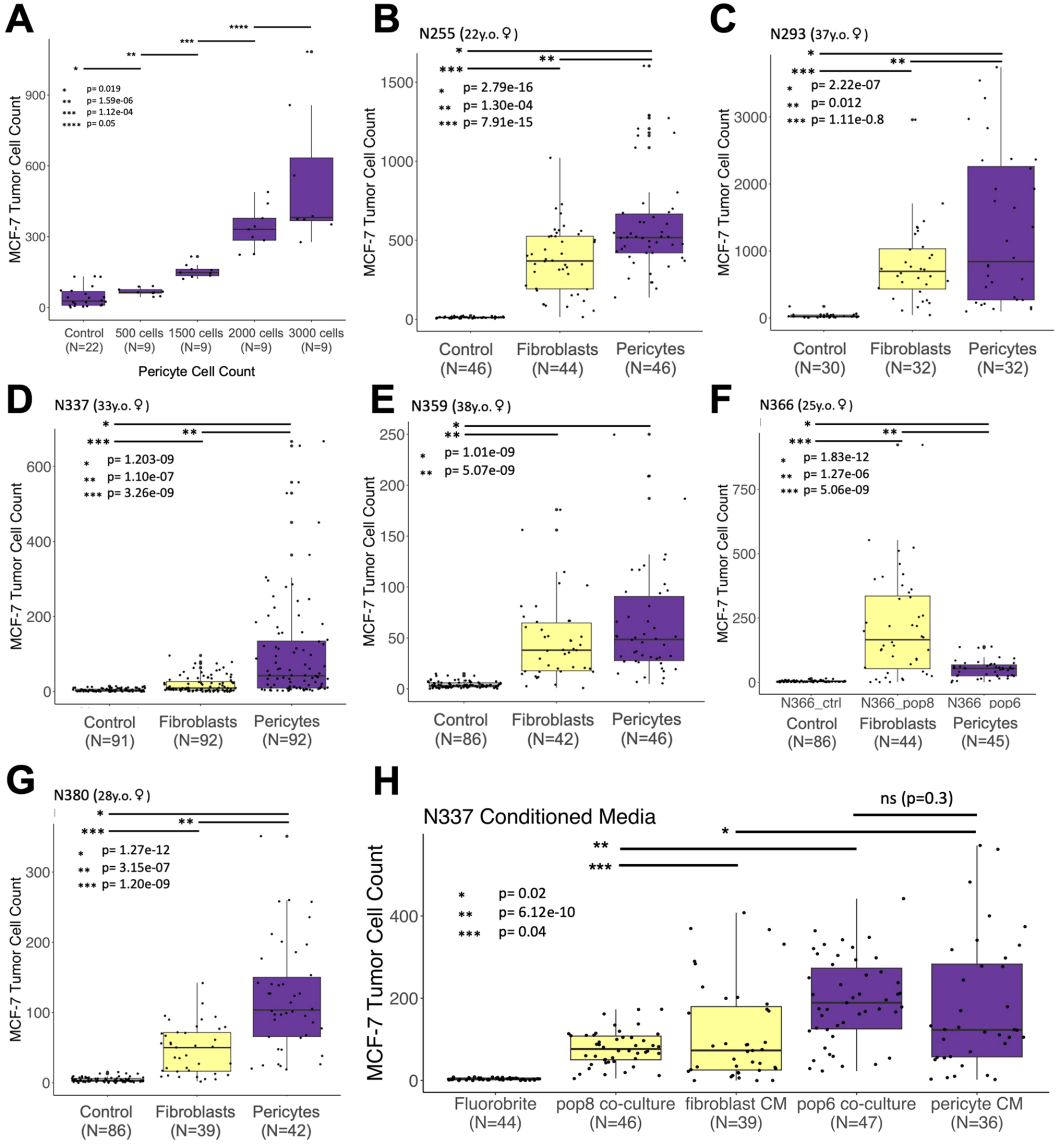
Stimulatory effect on tumor cells is a general feature of pericytes. **(A)** Counts of H2b-GFP-expressing MCF-7 breast cancer cells co-cultured with increasing amounts (doses) of pericytes (r^2^ = 0.869). **(B–G)** Counts of H2b-GFP-expressing MCF-7 breast cancer cells grown over two weeks after co-culture with irradiated breast primary cells (fibroblasts or pericytes) taken from seven different patient samples. **(H)** H2b-GFP-expressing MCF-7 cells co-cultured with fibroblasts/pericytes or cultured with fibroblast/pericyte-conditioned medium (medium refreshed every 2-3 days). Relevant p-values are indicated by *, **, and *** (calculated one-tailed t-test, unequal variance). The number of cells available dictated the number of replicates for all experiments. All experiments began with 50 tumor cells and were imaged after two weeks.

The above experiments were performed using pericytes from individual N274, with 1,500 pericytes accurately deposited by FACS into each well. At this seeding density, the pericytes have ample growth area remaining, meaning they were far from confluency. To determine the association between pericyte density and tumor cell stimulation, we seeded pericytes at different doses and repeated the experiment. We found the stimulatory effect was indeed dose-dependent. The final tumor cell counts correlated with the pericyte density (500-3,000 pericytes seeded per well, **Figure 7A**, r^2^ = 0.869). Each increasing dose of pericytes produced tumor cell counts that were statistically more significant than the previous dose (p-values ranging from 1.59e-6 to 0.05). These results suggest that the pericyte expansion we observed in tumors likely results in a more growth-stimulatory microenvironment.

To determine whether the tumor cell stimulation observed was a general feature of pericytes or unique to those from patient N274 used in the preceding experiments, we isolated breast pericytes and fibroblasts from six additional individuals (**Supplementary Figure 7**). Over two years, we created primary cultures and tested their ability to stimulate MCF-7 tumor cells (**Figures 7B–G**). Acquiring enough pericytes and isogenic fibroblasts to perform an experiment typically took approximately two months each (**Figure 6A**), and the number of replicates used in each experiment was dictated by the number of pericytes available (ranging between 32 to 92 replicates for each experimental condition). The results of these tumor co-culture experiments were conclusive: Pericytes produced a dramatic effect. Pericytes from every sample stimulated tumor cells and produced significantly greater cell counts than controls. Furthermore, as observed with N274, pericytes again stimulated and produced significantly higher tumor cell counts than isogenic fibroblasts in all but one experiment (**Figures 7B–G**). In these cases, the maximum observed tumor cell count from any fibroblast replicate never surpassed the maximum count observed in paired isogenic pericytes.

The above results demonstrated that pericytes, through direct contact or a secreted factor, stimulate tumor cell growth. To distinguish between these two possibilities, we tested the stimulatory activity of cell culture medium conditioned by pericytes. We discovered that the conditioned medium alone was sufficient in stimulating tumor cell growth, producing cell counts that were not significantly different than pericyte co-cultures (**Figure 7H**, p = 0.3). Furthermore, the stimulatory activity provided by pericytes, whether through co-cultures or conditioned medium, led to significantly greater numbers of tumor cells than those cultured with fibroblasts or medium conditioned by fibroblasts (**Figure 7H**). These results indicate pericytes stimulate tumor cells through an as-yet-unknown secreted factor.

## Discussion

The tumor microenvironment is a complex mix of elements that varies between patients, comprising not only tumor cells but also various stromal cell types, including perivascular cells (2, 38–40). Identifying the contributions of these different cell types on tumor development is fraught with challenges, given the intricate interactions and dynamic nature of the tumor microenvironment. Our findings highlight the significant role of pericytes within this context. We observed an increase in tumor cell proliferation in the presence of pericytes, their detachment from blood vessels, significant expansion and infiltration into the tumor stroma, and the association of their gene expression with poorer patient survival. These observations indicate that pericytes contribute to a supportive niche that facilitates tumor progression, which has important implications for our understanding of the different cellular elements in breast tumors and their contributions to disease pathology.

Establishing the perivascular contribution to different tumor pathologies has been challenging, and research on the pericytes’ role in breast cancer pathology has been relatively limited. Our focus on perivascular cells came only after our years-long cytometric and molecular dissection of the breast (25, 26), which produced a method to purify the different breast cell types –and a means to culture them. This comprehensive approach, using a panel of antibodies in combination, circumvented the oft-cited problem of pericyte identification (13, 14, 22–26). Leveraging these methods and the associated RNA-seq datasets of purified cell types allowed us to identify perivascular cells as potentially significant players in tumor biology. One of the first clues was the discovery that patients with tumors enriched with perivascular genes were nearly twice as likely to have died compared to those with lower perivascular gene expression (**Figure 3**).

This finding was particularly intriguing because other cell-type gene signatures, including those of endothelial cells—normally closely interacting with pericytes— showed no association with patient survival (**Figure 3**; **Supplementary Figures S1, S2**). A previous investigation that evaluated cell type-associated genes in breast cancer found a similar lack of prognostic value in their derived stromal-cell-specific gene sets (41). However, their stromal gene sets—like most in the literature (42, 43)—were not designed to resolve differences between fibroblasts, adipocytes, and perivascular cells—the three mesenchymal cell types composing breast tissues. Indeed, it is widely appreciated that cancer-associated fibroblasts (CAFs) are heterogeneous, not precisely defined, express different markers, and may have different cellular origins (44). This heterogeneity underscores the importance of accurately distinguishing between cell types. When we resolved these cell type differences, the relationship between tumors enriched in perivascular genes and patient survival was apparent. The biological basis explaining the association, however, was not.

The enrichment of perivascular genes in breast tumors could arise from different scenarios. We needed to visualize the perivasculature cells in tumors to explore these possibilities. However, existing methods for staining perivascular cells in tissues rely on markers that are either not universally expressed by all perivascular cells or expressed by other cell types—particularly fibroblasts, which have a similar morphology. This technical barrier and difficulty in discriminating between different mesenchymal cell types is widely appreciated (14, 20, 23, 24, 45).

To overcome this problem, we needed to identify a gene that is broadly expressed by all perivascular cells. It would also need to be differentially expressed in pericytes compared to fibroblasts. Our previous work and FACS strategy aided us again, which allowed us to purify breast pericytes from the other breast cell populations. Using this method, we sorted pericytes and subjected them to 10X single-cell RNA sequencing and analysis (**Figure 4**). As expected, multiple pericyte clusters were identified, along with the other cell types we included. In agreement with the criticisms in the literature, genes encoding commonly used pericyte markers were expressed in a manner that explained the critiques: Some were expressed by only a small fraction of the pericytes, others were limited to one or a few pericyte clusters, and some were also expressed by fibroblasts or other cell types (**Figure 4I**). Keeping these considerations in mind, we contrasted all pericyte clusters to fibroblasts, which provided us with a list of potential pericyte markers (**Supplementary Table 1**).

Immunostaining tissues for these markers revealed CGMP-Dependent Protein Kinase 1 (Pkg1) strongly expressed in perivascular cells along the blood vessel periphery, as well as being modestly present in the myoepithelium (**Figure 5A**; **Supplementary Figure S2A**). Notably, the *PRKG1* gene encodes two isoforms of cyclic GMP-dependent protein kinase (pkg1), a key mediator of the nitric oxide/cGMP signaling pathway, regulating smooth muscle contraction. Thus, the expression of Pkg1 by myoepithelial and perivascular cells is consistent with these cells’ physiological functions. We were not concerned with using this marker in tumor tissues, as myoepithelial cells are largely absent compared to normal tissues (46, 47). Nevertheless, co-staining breast tumors with EpCam did rule out an overlap with Pkg1 for all epithelial populations (**Supplementary Figure S5F**). Thus, identifying Pkg1 as a suitable marker provided a reliable antibody for staining and detecting perivascular cells in malignant breast tissues. Interestingly, after identifying Pkg1, we discovered a 1984 article that thoroughly explored Pkg1 expression in rat tissues (heart, intestine, diaphragm, mesentery) using light and electron microscopy (48). Our human breast data (scRNA-seq and immunostaining) is consistent with their results: all perivascular cells were Pkg1-positive, while endothelial cells and connective tissue fibroblasts were consistently negative. Our findings aligned with their conclusion that Pkg1 staining allows for studying all perivascular cells within a microvascular bed (48).

When we applied the Pkg1 antibody to breast tumors, we discovered that the results were similar to those in normal tissues: Pkg1 was present on pericytes and vascular smooth muscle cells (**Figure 5**). However, compared to normal tissues, Pkg1+ cells had expanded along blood vessels in nearly all tumors and extensively within the stroma in over half the samples (**Figures 5C–G**). These results identify perivascular cells as a major constituent of the tumor stroma in a subset of breast cancers. Due to their prevalence, these cells have likely been long misidentified as fibroblasts—or activated fibroblasts. Consistent with this conclusion are the results of a recent single-cell sequencing study of twenty-six breast tumors that identified the presence of perivascular-like cells (PVL) (49). Notably, in the pool of integrated tumor samples, perivascular-like cells constituted 45% of the mesenchymal cell population, including cancer-associated fibroblasts (CAFs) and PVLs. Analysis of their supplementary data revealed that the proportion of perivascular cells within the mesenchymal fraction of each tumor varied widely across the 26 tumors analyzed, ranging from as low as 10% to as high as 86% of all mesenchymal cells, which is consistent with our immunostaining results. Our observed increase in perivascular cell number and their migration away from the vasculature in tumors places these cells in close proximity to tumor cells, at an abundance that could affect tumor cell physiology and tumor progression.

Further investigation into the functional influence of pericytes led to a significant discovery: purified breast pericytes cultured ex vivo induced rapid tumor cell expansion. In pericyte co-cultures, tumor cell counts were significantly higher than those cultured alone or with activated fibroblasts (**Figure 6E**). Both luminal and basal tumor cell models were stimulated by pericytes, indicating that this stimulus could contribute to the pathology of different clinical breast cancer subtypes (**Figures 6F, G**). Furthermore, we found pericyte stimulation occurred in a dose-dependent manner (**Figure 7A**) and was a consistent feature across all seven of the primary pericyte cell lines tested (**Figures 7B–G**).

A striking finding was the different stimulatory activities observed between pericytes and fibroblasts. In six of seven experiments, pericytes produced significantly greater tumor cell counts than paired isogenic fibroblasts (**Figures 7B–G**). The exception was the sample from donor N366, where fibroblasts produced greater tumor cell counts. The reasons for this anomaly remain unknown. All pericyte lines were confirmed by CD36 immunostaining, and there were no notable differences in the donor’s age, race, FACS profile, or cell passage number (3p). Our single-cell analysis of uncultured pericytes revealed considerable heterogeneity in cells sorted directly from breast tissues. Undetected selection of specific pericyte subtypes in our cultures may account for the observed functional differences. Furthermore, while our samples showed consistency across samples, the limited sample size may not fully capture the diversity of pericytes across different patient populations, i.e., across the spectrum of breast tumor types. Larger, more diverse cohorts would help validate and extend these findings.

Finally, we discovered that the pericyte factor responsible for activating tumor cell proliferation is secreted. Pericyte cell contact was not required, as conditioned medium alone was sufficient to stimulate tumor cells (**Figure 7H**). These results suggest that the expansion of pericytes in breast tumors contributes to a local microenvironment that promotes tumor cell proliferation and pathology through a yet-to-be-determined secreted factor.

## Materials and Methods

### Breast tissues and primary cultures

Breast tissues from reduction mammoplasties were obtained from the Cooperative Human Tissue Network (CHTN), a program funded by the National Cancer Institute. All specimens were collected with patient consent and were reported negative for proliferative breast disease by board-certified pathologists. The University of New Mexico Human Research Protections Office granted use of anonymous samples through exemption status, according to the Code of Federal Regulations 45 CFR 46.101. Upon receipt, tissues were processed to organoids, as previously described (25, 26, 50). Briefly, tissues were rinsed in phosphate-buffered saline. Three approximately 1.5 cm³ pieces were cut away, embedded in OCT compound, and flash-frozen in liquid nitrogen for later cryosectioning. The remaining tissue was processed by slicing into smaller pieces and digesting for 12-18 hours in 0.1% collagenase I (Gibco/Invitrogen) in Dulbecco’s Modified Eagle Medium (5% pen/strep, 1% normocin) with gentle agitation at 37°C. The resulting organoids were collected via centrifugation (100g for 2 minutes) and either archived in liquid nitrogen (90% FBS, 10% dimethylsulfoxide) or further processed for flow sorting.

### Cell lines

MCF-7 and MDA-MB-231 cell lines were obtained from the American Type Culture Collection (ATCC). Both cell lines were cultured in Dulbecco’s Modified Eagle Medium (Sigma D5796) containing 100U ml^-1^ penicillin, 100 mg ml^-1^ streptomycin, and 50 mg ml^-1^ Normocin.

### Antibodies

A list of antibodies and reagents used in this study is provided in **Supplementary Table 2**. The table includes antibody clone designations, conjugations, and supplier product numbers.

### Survival analysis

Survival analysis was performed using the top twenty most uniquely expressed genes in each breast cell type. Briefly, differential expression analyses was performed using DESeq2 (51) on RNA-sequenced transcriptomes of FACS-purified cell types to obtain rlog transformed values (regularized log transformation), using data from Del Toro et.al (26). We analyzed every transcript, in each breast cell type, across every cell-cell comparison to identify the genes expressed above a given threshold setting. Using a stringent rolling threshold, we identified the twenty genes that were most uniquely expressed by each breast cell type.

Survival analysis for each cell-type-specific gene set (consisting of the twenty genes identified from our RNA-sequencing analysis) was performed using Survival Genie (52), filtering for samples within the 25^th^ and 90^th^ percentile of expression of the TCGA breast cancer dataset (29). Survival Genie uses the survfit function (R-studio) to estimate the overall survival/event-free survival ratio and performs a log-rank test to compute differences in overall survival/event-free survival ratios between the defined high- and low-risk groups (tumors with either high or low expression of cell-specific genes). Univariate analysis with Cox proportional hazards regression model with Wald test is performed on the patient data using coxph function in R/Bioconductor. The p-value from the log-rank test and the hazard ratio from the Cox proportional hazards regression model are reported in the figures.

### Cell sorting

Pericytes from normal breast tissue were FACS-isolated using previously described methods (25). Briefly, cell suspensions were prepared by rinsing tissue organoids (from collagenase digest) in PBS, then pelleting them via centrifugation at 100g for 2 minutes. After removing the PBS by aspiration, the organoids were suspended in 1 ml of Cell Dissociation Reagent (Sigma #C5914 or Thermo #13150016) and incubated for two minutes at room temperature. Then, 3 ml of 0.25% trypsin (Thermo #25200072 or TrypLE) was added. Samples were incubated in hand, at body temperature, allowing for visual inspection and brief (1-3 second) pulse vortexing every 30-60 seconds. When the mixture became cloudy due to the dissociating cells (about 8-10 minutes), the organoids/cells were gently pipetted through a 16-gauge needle until clumps dissipated.

Next, cell suspensions were filtered through a 100 µm cell strainer and 3 ml of 0.1% w/v soybean trypsin inhibitor (Sigma #T9128) was added to stop the digestion. The suspensions were then filtered through a 40 µm cell strainer, rinsed with 10-20 ml PBS, and pelleted by centrifugation at 400g for 5 minutes. The cells were rinsed in 10 ml Hanks’ balanced salt solution/1% BSA (w/v), counted, and pelleted again by centrifugation. After the final centrifugation, nearly all of the Hanks/BSA was aspirated, leaving the cell pellet in roughly 60 µl of Hanks/BSA. The pellet was resuspended in this small volume, and FACS antibodies were added (CD45, CD49f, CD24, Muc1, Podoplanin, Thy1, CD34, CD10). Samples were incubated on ice, covered, for 30 minutes. Following incubation, the cells were rinsed in Hanks/1% BSA, centrifuged (400g for 5 minutes), and resuspended in Hanks/BSA with To-Pro-3 viability marker (diluted 1:4000, Thermo T3605). The suspensions were then filtered through a 40 µm cell strainer cap into FACS tubes. Samples were kept chilled on ice throughout the procedure. FACS was performed using a Sy3200 Sony Sorter. For cell cultures, pericytes were sorted three times to ensure purity.

### Single-cell RNA-sequencing

For single cell RNA sequencing analysis, pericytes from a reduction mammoplasty (23-year-old female) were FACS sorted twice. We did not perform a third and final ‘purity-sort,’ so that residual epithelial cells, endothelial cells, and fibroblasts would remain –and could be used as internal sequencing controls. The sequencing library was prepared using the Chromium Next GEM Single Cell 3’ Reagents Kit v3.1 (10X Genomics; Pleasanton, CA). The library was sequenced at the University of Colorado Denver, Anschutz Medical Campus. The raw data was aligned and prepared at the Analytical and Translational Genomics Shared Resource at the University of New Mexico Cancer Center using Cell Ranger™ Software according to the manufacturer’s guidelines. We performed the downstream analysis in R-studio using Seurat Bioconductor package (53).We performed the downstream analysis in R-studio using Seurat Bioconductor package (53).

### Tissue immunostaining

Immunofluorescence was performed on 10 μm cryosectioned tissue sections as described previously (25). Briefly, tissue sections were fixed with 4% paraformaldehyde for 5 minutes at 23°C, followed by 4% formaldehyde/0.1% saponin for another 5 minutes at 23°C. The tissues were then rinsed three times (20-30 minutes total) in wash buffer (0.1% saponin/10% goat serum in PBS) and incubated with primary antibodies, typically overnight at 4°C. Following the primary incubation, samples were washed and incubated with secondary antibodies conjugated with Alexa Fluor 488 and 594 (Thermo). After a 1-hour incubation at 23°C, samples were rinsed in PBS, and their nuclei counterstained with 300 nM DAPI (4’,6-Diamidino-2-Phenylindole, Dihydrochloride, Thermo). Coverslips were mounted with Fluoromount G (Southern Biotech). Images were captured using the EVOS FL Auto imaging station. If needed, Photoshop was used to increase image contrast (across the entire image) and annotate the images with the antibodies used for staining.

### Tumor cell co-cultures

Tumor cells (MCF-7 or MDA-MB-231) were co-cultured by seeding them into individual wells of 96-well culture dishes containing collagen I (Purecol), primary pericytes, or primary fibroblasts. Pericytes and fibroblasts were seeded into the 96-well dishes at a density of 1,500 cells per well, followed by irradiation with 30 Gy X-ray and incubation overnight at 37°C. The medium was then replaced with Fluorobrite medium supplemented with 1% filtered FBS (containing 100 U/ml penicillin, 100 µg/ml streptomycin, and 50 µg/ml Normocin), which was refreshed weekly or as indicated in the figure legends. H2b-GFP MCF-7 cells or H2b-GFP MDA-MB-231 cells (54) were then sorted into replicate wells using a 14-channel SONY SY3200 sorter at densities of 50 cells per well. The plates were scanned on day 14 using the EVOS scan function, and the GFP-labeled tumor cells were quantified as previously described (55).

### Conditioned medium assay

The conditioned medium experiments were performed similarly to the co-culture experiments. Briefly, pericytes or fibroblasts were grown to confluency. Upon reaching confluency, the medium was switched from M87 (10% FBS) to Fluorobrite (1% FBS), and the cells were incubated for 2-3 days. After this incubation, the conditioned medium was collected, filtered, and added to 96-well plates containing pre-seeded H2b-GFP MCF-7 cells (50 cells/well). Controls included tumor cells incubated with: a) medium alone (Fluorobrite/1% FBS), b) irradiated fibroblasts, and c) irradiated pericytes. After 14 days, the plates were imaged, and cells were quantified as in the co-culture experiments.

## Data Availability

The data presented in the study are deposited in the OSF repository, accession number DOI 10.17605/OSF.IO/U9RVK.
