# Supplementary material for: Breast pericytes: a newly identified driver of tumor cell proliferation
Source: Front Oncol. 2024 Dec 17;14:1455484. doi: 10.3389/fonc.2024.1455484 (PMC11685225; doi:10.3389/fonc.2024.1455484)
Supplement: Supplementary file 4 [file DataSheet1.pdf]

Supplementary Figure S1

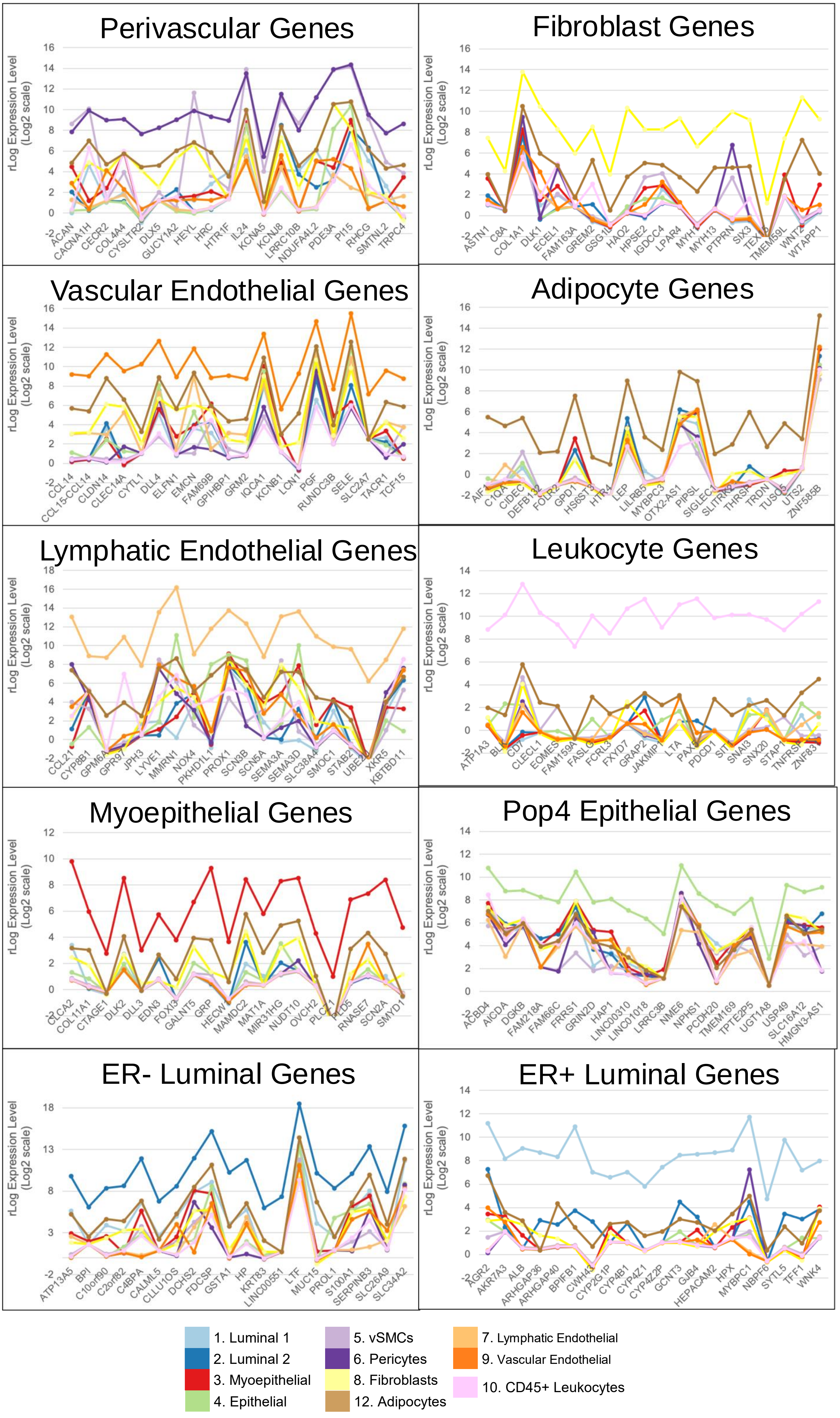

Supplementary Figure S1: rLog mRNA expression values for the top twenty most unique genes expressed by each purified breast cell type (RNA-sequencing of FACS-purified breast cell types, from Del Toro et al.)

Supplementary Figure S2

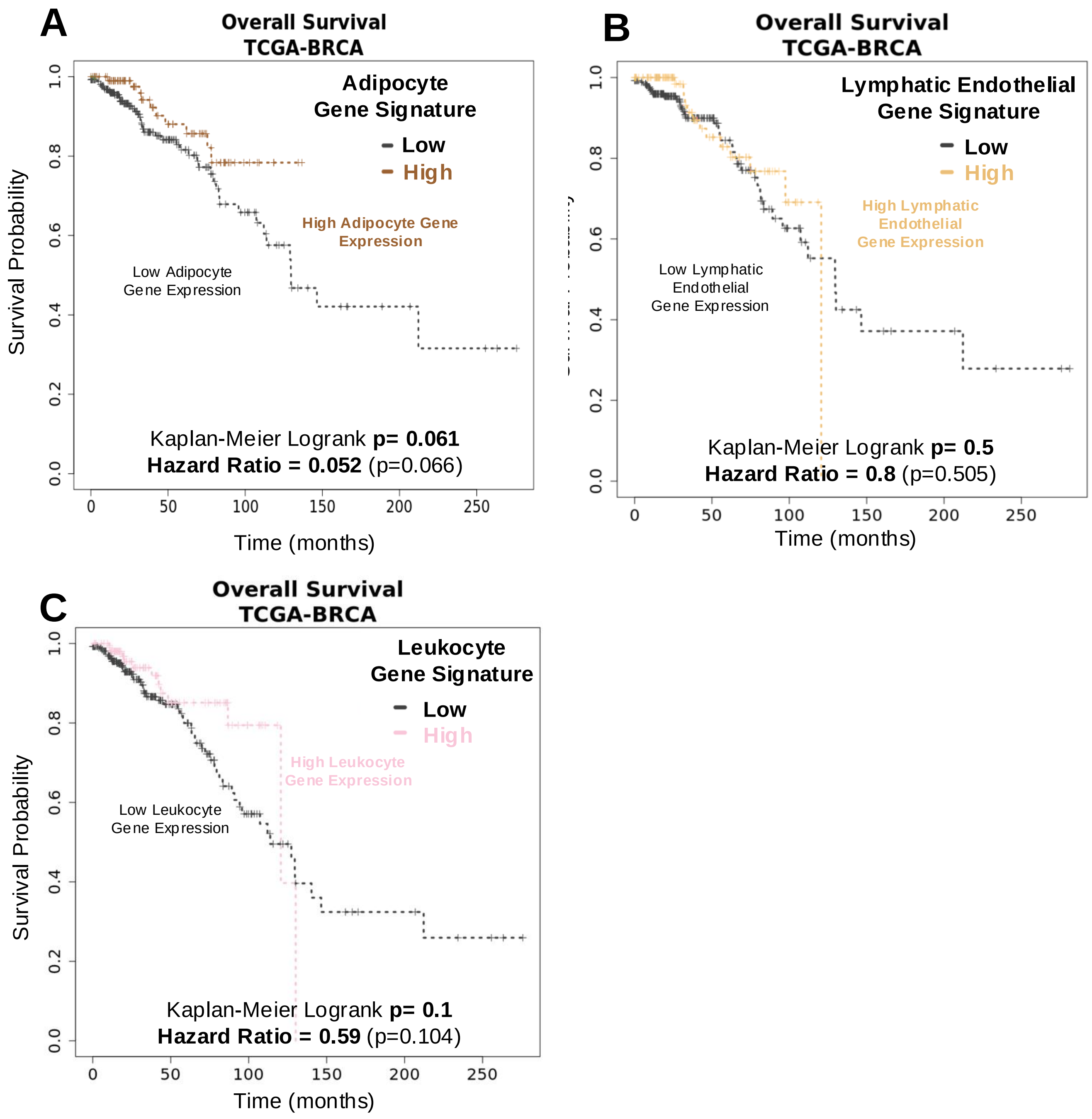

Supplementary figure S2: Kaplan-Meyer curves of breast cancer survival (TCGA- breast cancer data set) associated with genes unique to breast a) adipocytes (logrank: p= 0.066, HR=0.052), b) lymphatic endothelial cells (logrank: p=0.505, HR= 0.8), and c) leukocytes (logrank: p=0.104, HR= 0.59). Adapted from SurvivalGenie.

Supplementary Figure S3

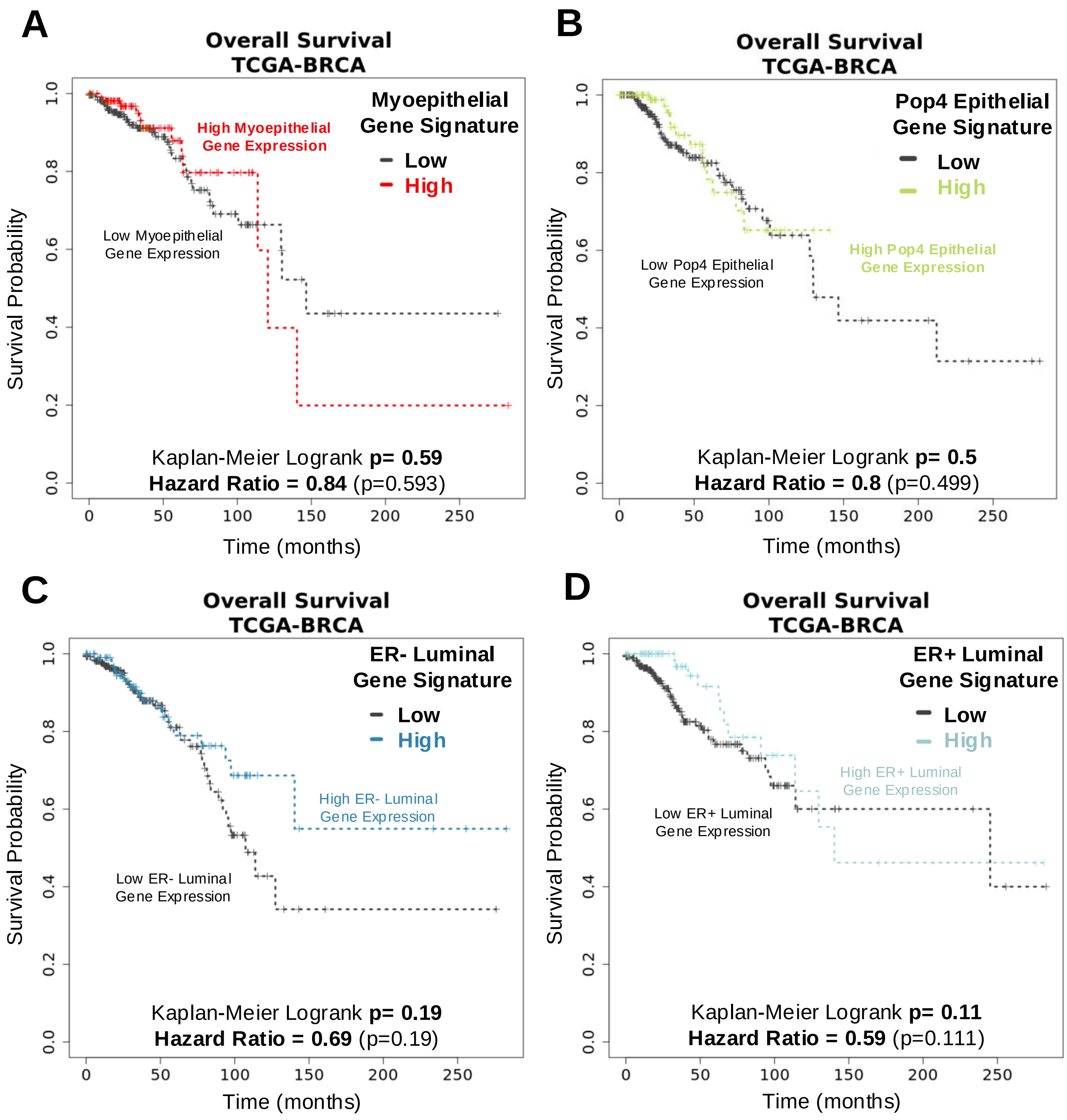

Supplementary Figure S3: Kaplan-Meyer curves of breast cancer survival (TCGA- breast cancer data set) associated with genes unique to breast a) myoepithelial cells (logrank: p= 0.593, HR=0.84), b) pop4 epithelial cells (logrank: p=0.499, HR= 0.8), c) ER- luminal cells (logrank: p=0.19, HR= 0.69), and d) ER+ luminal cells (logrank: p=0.111, HR=0.59). Adapted from SurvivalGenie.

Supplementary Figure S4

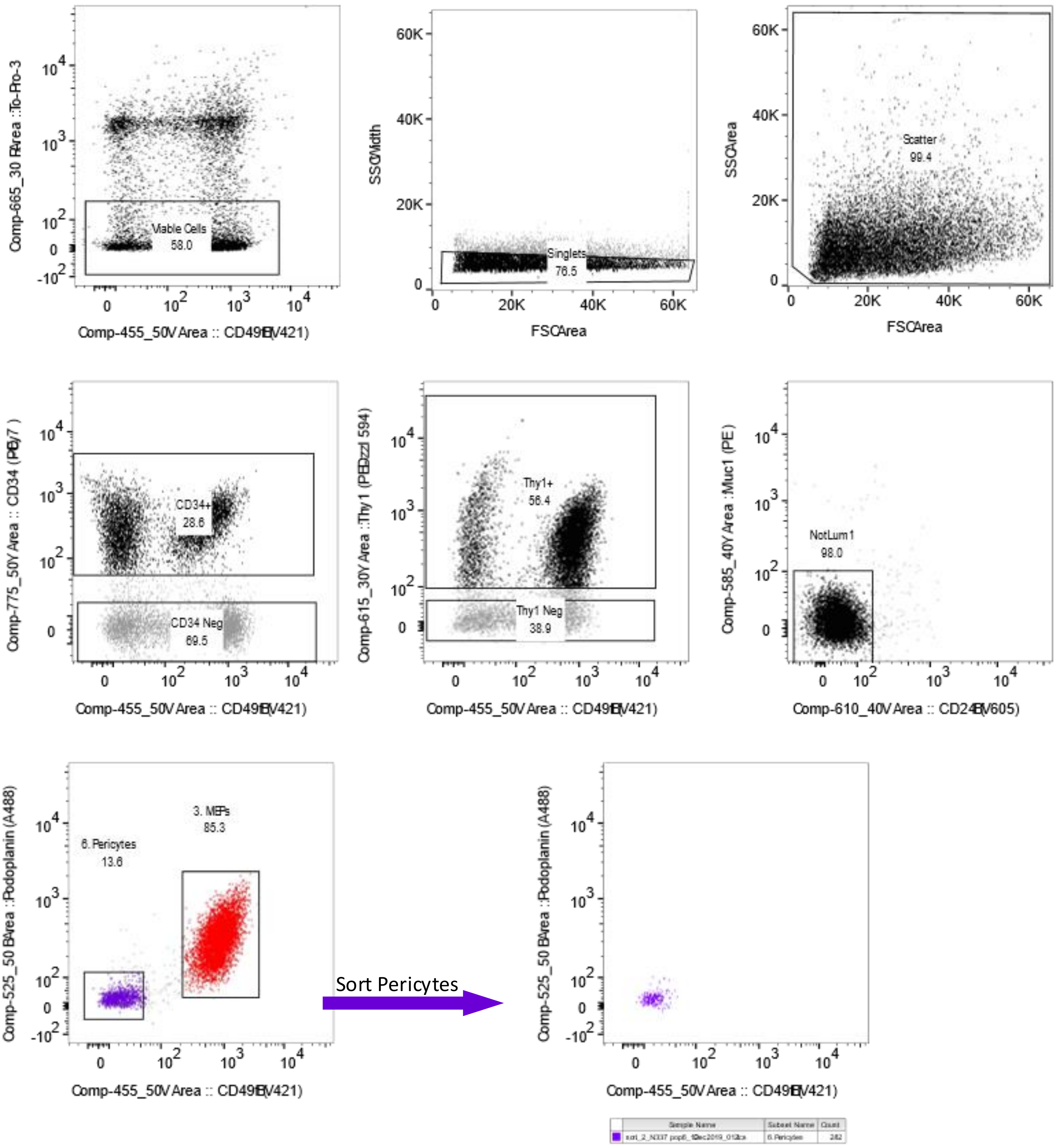

Supplementary Figure S4: FACS gating strategy for purifying the pericyte population from normal breast tissue (33-year-old white female).

Supplementary Table 1: Exported list of genes from single-cell data analysis containing markers differentially expressed between grouped pericyte and fibroblast clusters. The statistic pct.1 indicates the fraction of perivascular cells that express a given gene and pct.2 indicates the fraction of fibroblast cells that express the given gene. The markers were determined using the ‘FindMarkers’ Seurat function.

Supplementary Table 2: Antibody clones used for immunostaining and FACS.

Supplementary Table 3: Breast tumors with associated perivascular expansion grading, histological grading, and subtypes (determined by ER/PR/Her2 positivity). * Scarff-Bloom-Richardson grading convention † Elston and Ellis modification of Scarff-Bloom-Richardson Grading System ‡ Nottingham score § Performed on biopsy but was not reported in pathology report

Supplementary Figure S5

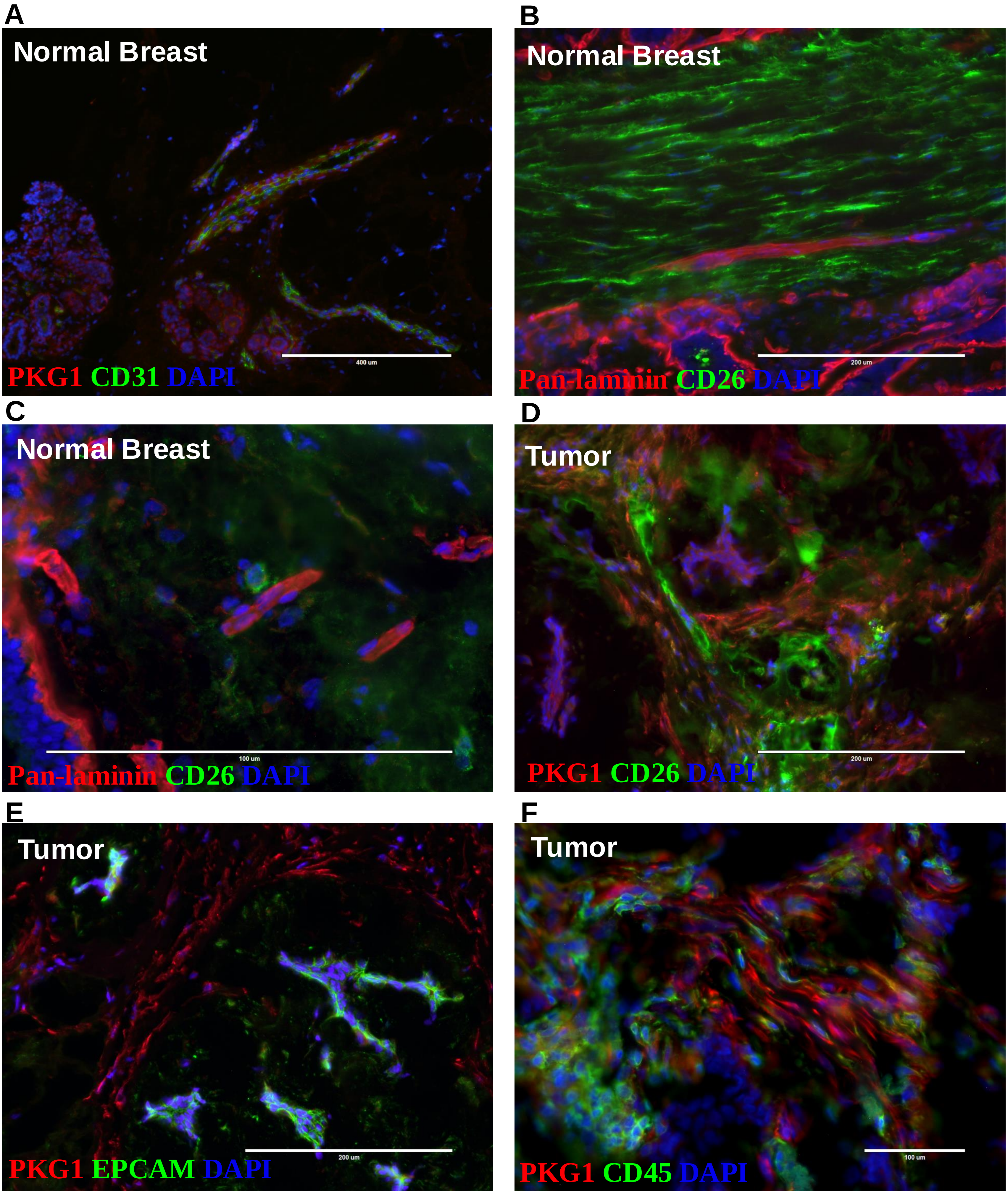

Supplementary Figure S5: Representative immunostaining of normal breast tissue with a) PKG1 (red) and CD31 (green), b-c) Pan-laminin (red) and CD26 (green). Representative immunostaining of invasive breast tumors with d) PKG1 (red) and CD26 (green), e) PKG1 (red) and EPCAM (green), and f) PKG1 (red) and CD45 (green).

Supplementary Figure S6

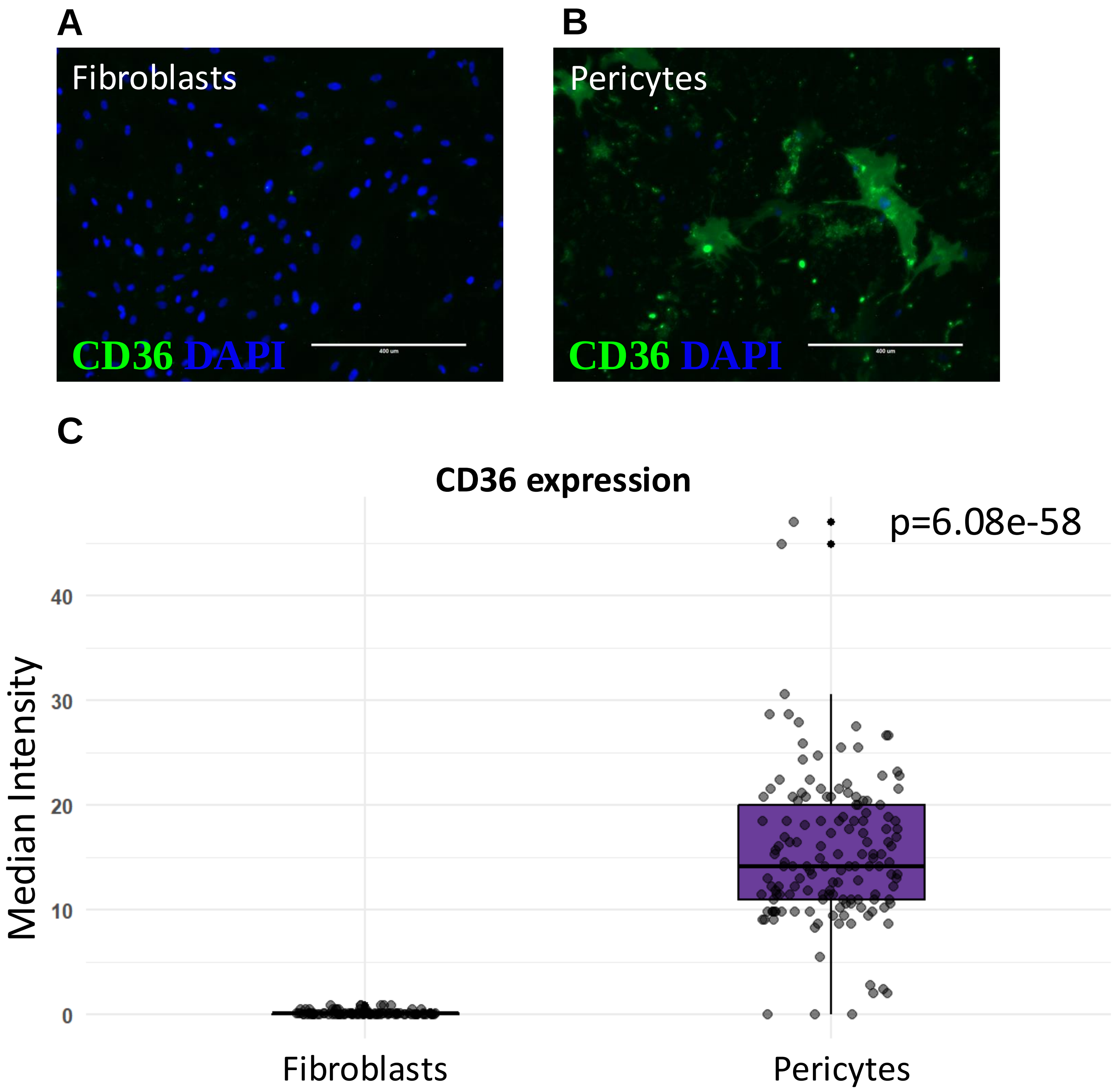

Supplementary Figure S6: Normal breast immunostaining a) fibroblasts and b) pericytes with CD36 (green)– obtained from 25-year-old black female. c) Quantification of CD36 expression in normal breast fibroblasts and pericytes (p=6.08 e-58).

Supplementary Figure S7

Sample	Sex	Age	Race
N337	F	33	W
N274	F	37	W
N255	F	22	W
N359	F	38	W
N380	F	28	W
N293	F	37	B
N366	F	25	B

Supplementary Figure S7: Sample demographics including sex, age, and race.
